# Indiana Association of Veterinary Graduates

**Published:** 1891-02

**Authors:** H. R. Macaulay

**Affiliations:** Secretary


### SOCIETY PROCEEDINGS.

*Indiana Association of Veterinary Graduates.*—The Annual meeting of the Indiana Association of Veterinary Graduates was held in Indianapolis on January 7th and 8th.

On the evening of the 7th the meeting was called to order at 8 P. M., neither the President nor Vice-President were present, Dr. Macaulay was called upon to take the chair until after the election of officers for the coming year.

The minutes of the previous meeting were read and approved and after preliminary business had been attended to the election of officers took place, with the following result:

For President, Dr. J. Rodger, of Anderson.
" Vice-President, Dr. G. Ferling, of Richmond.
" 2nd " Dr. J. Culbert, of Portland.
" 3rd " Dr. G. Buckner, of Rockville.
" Secretary, Dr. H. Macaulay, of Indianapolis.
" Treasurer, Dr. E. Diggs, of Winchester.

The newly elected President, Dr. J. Rodger now took the chair and the first paper of the meeting was read by Dr. E. Digg on rheumatism. He considered it an acute febrile disease brought on by obscure climatic and diatetic influences, and characterized by pyrexia sweats and acute shifting inflammation of the joints and other structure. The essayist places the greatest stress on the heredity of the trouble and says that 27 per cent. of all cases can be traced to that cause. Of the other great causes the most important are exposure to cold and wet, and it is frequently a sequence of influenza.

Dr. Diggs then went on to show the anatomical changes found in diseased joints, but believed that when death occurred from rheumatism it was due to complications with the organs, particularly heart, pleura or lungs. If there has been hyperpyrexia before death the blood is fluid for a considerable time after, and the liquor sanguinis is alkaline and there is no increase in uric acid—lactic acid.

In treating of the pathology of the disease the essayist mentioned it was very obscure, but gave the theories advanced by a number of eminent authorities, the majority of whom give lactic acid as the chief reason some others say it is due to a germ, etc. Dr. Diggs expressing his belief that it was not due to a micro organism. He then detailed some cases he had had in practice; his treatment in such cases being nitrate of potash—salicylate of soda and valerianate of morphine when necessary.

Dr. Thompson inquired of essayist how he distinguishes between acute rheumatism and osteo porosis? The questionsr saying he had had many cases where he had found it impossible to distinguish between the two excepting by an examination of the urine. If urine was acid it was rheumatism, if alkaline osteo porosis.

Dr. Culbert.—Had Dr. Diggs ever noticed any cracking of joints No. Did he not think hot water very beneficial in cases where joint was effected? Yes, and essayest often used in cases of severe pain in a joint a liniment of aconite, belladonna and camphor with oil.

Dr. Ferling believed in the beneficial effect of hot water, as he could speak from experience, being a martyr to rheumatism.

Dr. Macaulay in his treatment always gave brisk cathartic and followed this generally with nitrate of potash and soda salicylate. Had had two cases of osteo porosis during the year, but had no difficulty in diagnosing; believed that in rheumatic cases the pain and lameness came on suddenly, while in osteo porosis the lameness was gradual and constantly increasing, and not so inclined to shift.

Dr. Roberts does not believe in large doses of sal soda, but prefers to give ʒss doses frequently and in combination gives diuretic and aconite.

Dr. Thompson mentioned that he had never seen a case that occurred where a horse stood on a board floor, and believes that the damp earth was frequently a cause of the trouble.

Dr. Rodger never gave aloes in rheumatism, but preferred sulphate of magnesia, followed by nitrate of potash and colchicum.

Dr. Sheffer believed in good hygenic surroundings while treating.

The meeting then adjourned until 9 o'clock the following morning, when it was called to order, the President, Dr. Rodger in the chair.

Dr. Macaulay then read his paper on "Counter irritation versus cold applications in Pneumonia."

The essayist took up the nature of pneumonia so that the effects of the two applications might be the more thoroughly understood. The starting point in pneumonia occurred when the animal was said to "chill," the chill being of nervous origin and defined as "a prolonged depression of nerve force without the reaction which should occur immediately after the collapse." In the case where a "chill" occurs the action of cold air on the cutaneous nerves has been so severe that the afferent impulses have for a time paralyzed the nerve centres so that control is lost over the vaso-constrictor centre and the consequence is the caliber of the blood-vessels is constricted and less blood is carried in them. This condition of affairs is followed by the "re-action" when the nerve centres recover from the shock and reassert command over the vaso-constrictor centre so that the blood again flows freely through these vessels that were constricted, and it is here the crisis occurs. If the action of the cold has not been too depressing in its effects the minute nerves once more take command over the capillaries and all goes on well. If, on the other hand, the depressing effect has been too severe these nerves are not able to assert command over the walls of the capillaries, these then distend overmuch and fill with blood and we have what we term congestion, and when the congestion is in the lungs it is followed by inflammation. Dr. Macaulay then mentioned other changes noticed with the inflammation, and in speaking of the increased heart beat and increased respiration, while believing that the first was partially due to lack of resistance of blood pressure and the second to less lung structure having to do more work, yet was sure if the result of these actions on the diseased part was studied, naturewould be seen trying to rectify things by forcing the blood through the congested vessels with the increased heart beat and the increased respiration by contraction and expansion of lung helping the heart by mechanically forcing the blood along.

The physical symptoms of pneumonia were then spoken of and then the action of counterirritants on the disease.

The first action of the counterirritant on the nerves causes a constricting of the vessels, this being quickly followed by enlargement and engorgement with blood. Up to this point the essayist remarked that the results of the action of the cold and of the blister had been very similar. The pain caused by the counterirritant was then dealt with, the essayist mentioning that pain must be due to injury of the nerves, and continuing referred to an able paper by Dr. White, lecturer at Guy's Hospital, London, in which all pyrexias were believed to be of a nervous origin. Dr. Macaulay believed that the pain must necessarily increase the temperature and aggravate the disease. True, there was an external "reservoir" formed for the blood, but the pain caused by the counterirritant was shown by great uneasiness of animals, but that when the intensity of the pain has worn off, the animal becomes stationary and moves the area over which the counterirritant is as little as possible; the consequence being that the upper part of lung does more work, while lower or diseased part does less, and because of this the blood stagnates much more readily, and the pain is thus making the lung exactly contrary to nature's dictations. The enforced rest caused by a counterirritant the essayist believed to be of great benefit in a great number of cases, but the lungs were organs that had to go on with their duty day or night, and whatever tended to interfere with that function aimed directly at life of animal. The essayist believed many an animal recovered that had been blistered, but that the recovery was in spite of the blistering.

At this point the essayist compared the treatment of pneumonia of bygone days and the treatment of to-day, referring to the difference in blanketing and keeping room close and hot, but more particularly to the change in the administration of drinks. Formerly water had to be warmed, now it was cold; the demands of nature, as seen by the patient's desires, being attended to. The hot and cold water must be much the same in their compositions; if anything the heated water was purer, and this being so, the difference in their action on the patient must be purely mechanical, the cold reducing the animal's temperature much the same as a cup of cold water would reduce the temperature of a basin of warm into which it had been poured. When looked upon in this light the essayist thought it forcibly suggested the use of cold water externally, the action of a blanket wrung out of cold water and applied to the chest and covered with a dry blanket would be two-fold. The first action of the cold on the nerves would be the same as the blister, there is a contraction of the blood vessels, followed when the reaction comes by the distention of the capillaries, and secondly, the mechanical effect of the cold blanket drawing the heat from the body until all is warm. The essayist believed it could be done a number of times, the change of blankets being expeditiously done and the thermometer guiding when to stop.

The Pneumo-cocci bacillus is put down as the cause of the trouble very frequently, and essayist believed it frequently could be found especially in cases following influenza but even here the cold water by reducing the temperature was beneficial, as it is well known the bacilli thrive more heartily when the temperature is higher than normal;

Discussion followed: Dr. Thompson thought cold applications contra indicated as cold had been the primary cause of the disease, enquired of essayist if he would take a patient with a temperature of 106° and lead him to the street on a cold day, so that patient might have beneficial effect of cold wind to lower his temperature, believes that if a little cold would do good in a fever, considerable cold should do more. Answer. The cases are in no way parallel. In the one the application of cold in the form of a wet blanket, covered with a dry one, the amount of cold is limited and the bulk of animals body in its heated condition will heat the wetted blanket. The shock here cannot be so severe as to cause a chill when the reaction occurs. By using the cold to side we are nearly following out nature's promptings when she caused the patient to crave for the cold water rather than the hot.

Dr. Culbert.—Thinks the cold good, but to facilitate the changing of blankets or do away with the changing entirely, would recommend the use of bags such as Dr. J. Mager's patent.

Dr. Diggs.—Does essayiest consider the use of cold applications to be of benefit in congestive stage? Yes, when temperature is high.

Dr. Roberts.—Does not favor use of blister. It prolongs case that might be cut short. Considers blistering merely a way to make a large bill on owner.

Dr. Culbert.—What internal treatment do you recommend? Ans. Any antepyretic medicine. Quinine. Acetanilde, Sal-soda, potash nitrate, etc., should work well in conjunction with cold-applications.

Dr. Shaffer.—Never used any external applications but believes with Prof. Williams that in stagnating cases, blisters by rousing the whole system might be beneficial.

Dr. Thompson.—Believes blister to be of service where pleura is affected but not when lung substance itself is.

Dr. Culberts.—Only objection to the cold water treatment is that we have not as good surroundings for our patients as medical practitioners, regarding blistering does not think he has ever killed any with it but certainly believes he has prolonged cases.

The meeting was adjourned until after dinner and met again at half-past one. Dr. M. Knowles in a short address moved, Dr. A. Thompson seconded, that the following resolutions be passed.

*First.* That Dr. F. Billing's be made an honorary member of this Association.

*Second.* Resolved that it is the sense of the Indiana Association of Veterinary Graduates that the investigations of Dr. F. S. Billings on contagious and infectious animal diseases have been the only investigations of merit made in these United States.

And be it further resolved that we feel indebted to Dr. Billings and the State of Nebraska for these investigations. And be it further resolved that since the State of Nebraska had Dr. Billings in her employ at the time these investigations were made and that in our belief Dr. Billings can give the Agricultural and Scientific world farther enlightenment on infectious and contagious diseases, we therefore request the State of Nebraska to re-employ Dr. Billings for said investigation.

Be it further resolved that a copy of these resolutions be sent to Dr. Billings and the State University of Nebraska, and that they be spread on the minute book of this Association.

These resolutions were passed unanimously and it was just at this moment Dr. Macaulay arrived. and at the request of Dr. Knowles the President read the resolutions over for his benefit.

Dr. Macaulay was sorry he had been detained so as to have been unable to have been present when these resolutions had been moved, as he considered it was something with which we as a Society should have had nothing to do. He understood that Dr. Billing's views on many subjects, were at variance with a majority of our leading veterinarians, and by siding with Dr. Billings in this case we were making ourselves antagonistic to them. If any such set of resolutions were to come before the association he believed they—the members— should have been warned of it in order to satisfy themselves that if they took any steps at all it would be in the right direction. The resolutions were passed and his protest he knew amounted to nothing, but he made it nevertheless.

Dr. Bell was then called upon to read his paper on "Rare Cases in Practice," and reported a case of Tenotomy in a colt, 4 months old, that had contracted tendons in all four legs, the right front one being so much contracted that the colt's toe only touched the ground, the other three were also much contracted but not so much as the right. Dr. Bell made his case very interesting by having cuts of all the different positions the legs were in. On the right front leg the incision was made half way between knee and fetlock. and both perforatus and perforaus were divided in the usual way, anteseptic precautions being taken, and cut after operation being filled with Quinine Sulphate, a pledget of cotton, and leg lightly bandaged. This made colt almost bring fetlock to ground.

Four weeks after first operation the left foreleg was operated on using no antiseptic precaution but injecting a four per cent solution of cocaine. As this leg was not so much contracted the perforans only was severed, and on stretching the leg forward the cut edge of the tendon separated about one inch. In this case the wound was dressed with Quinine Sulphate. On being let up the colt's foot came down in a natural position. In all cases the heels were lowered before operating and toes left long. Both the hind legs were also operated on without any antiseptic precaution, and although one was not bandaged there was no difference noticed in their healing.

The first operation was during the latter part of August and at present the colt is sound on three legs, but still somewhat down on the right foreleg, but a good recovery is expected since that leg is now in a shoe made expressly for it.

Dr. Thompson believed the right front leg would have done better if left entirely alone after the operation, he has had such cases and they ultimately came to natural position. Believes it is proper in such cases to raise heel rather than cut it down.

Dr. Knowles.—Believed that if right front leg had been let alone it would have come all right in time.

Dr. Roberts.—Had had three cases of cut tendons—one accidently cut—died. His second case was in a large draft horse. After operation a shoe such as Dr. Bell describes was applied and in six weeks horse was at work. In third case no shoe was put on and foot went down and stayed down.

The essayist mentioned here that the colt had not been born with contracted tendons for legs were natural and puffy the week before contracting.

He also uses quinine in all new cuts as in cases treated that way there is not one tenth amount of suppuration, and wounds generally heal by first intention.

Dr. Thompson now read his paper on "Administration of Medicine."—The essayist first impressed upon his hearers the necessity of the purity of the drug employed—especially when we know there are so many adultera. tions. The strength of the drug we use is also of vital importance, and Dr- Thompson believes fluid extracts should in all cases be used in preference to tenclures, as tenclures vary in strength in every different drug house. The administration of medicines in bolus form the essayist considers very unsatisfactory and especially so when chloral is to be given, he knowing of two cases where a capsule so given lodged in the fauces and dissolved there, death resulting in both cases—believes many gelatine capsules never dissolve in the horse at all and pass out as administered.

The quantity of medicine administered should always be minimum dose for desired effect, and in this respect practitioners are not careful enough, and also considers it wise for the practitioner to attend to the actual giving of medicine as possible and not to trust to grooms. In cases of Indigestons or Colics, the essayist says, it must be remembered that absorbtion is very slow and it is therefore better to give medicine hypodermically or intravenously. In any case where liquids are to be given use hard rubber syringe. Dr. Thompson considers Enemas almost valueless.

The essayist has had good results from tapping in cases of flatulent colic and injecting into bonel per camula from four to six ounces of glycerine, believes it stops short formation of gas.

Believes that only in Tetanus is administration of medicine per rectum preferable to other methods, and in this case form your medicines into small suppositories and insert by means of long dressing forceps.

Dr. Bell does not see how so large a thing as a capsule can pass through the pyeoric orifice of stomach unless digested or dissolved.

Dr. Bell.—How do you give aloes? In solution.

Dr. Macaulay.—In giving medicine two things have to be considered, the man and the patient. Believes the quieter we can give medicine say to a feble patient the more good it will do as the patient is not excited and does not fight, for this reason always use a hand rubber syringe. Wheu properly administered the animal takes it quietly and the giver is not besmirched as by bottle. Has never found Aloes and Calomel together dangerous.

Dr. Roger now read his paper on Indigestion.

The essayist enumerated the causes of the trouble—dentition sharp molars—inferior food. In young animal form method. Believes more common in draught-stock as stomachs in these are smaller than in many lighter breeds and in these heavier horses there is more impaction and these cases generally are fatal. The symptoms were chiefly shown by eructations of gas, quicker breathing and pain.

In treatment essayist is very fond of Pilocarpine, injections into trachea in from 1 to 2 grain doses, considers that in this dose it acts as a narcotic, depressant and cathartic also likes the action of Linseed oil, Turpentine and Aqua Ammonia, does not care for morphine because of its stopping the action of abdominal viscera. Uses enemas to hurry action of bowels.

Dr. Shaffer.—Does essayist ever use Pilocarpine with Eserine? No, because sweats are already profuse enough, combines Eserine with Atropia.

Dr. Feiling.—Does horse ever vomit and recover with you? No. Dr. Feiling relates a case where the regurgetation took place and animal was well and eating hay in half an hour.

Dr. Macaulay.—Very partial to Pilocarpine Charcoal and Sal Soda when eructations are present. Never had used Eserine in cases of indigestion but in cases where it was used always combined Pilocarpine as it appeared to lessen the severe griping of the eserine alone.

Dr. Culbert.—Does essayist give Eserine when animal is weak? No, it is too depressing. If there is no response to eserine give oil.

Moved by Dr. Feiling, seconded by Dr. Diggs, that the next meeting of the Association be held at Richmond in June. The date yet to be decided on.

The President then named essayists for next meeting.

The meeting then adjourned.

H. R. MACAULAY, *Secretary.*

